# Novel Technologies in Preterm Birth Prediction: Current Advances and Ethical Challenges

**DOI:** 10.34763/jmotherandchild.20252901.d-24-00048

**Published:** 2025-05-24

**Authors:** Marzhan A. Kassenova, Alma-Gul’ R. Ryskulova, Mairash A. Baimuratova, Tatyana M. Sokolova, Assel K. Adyrbekova, Indira S. Yesmakhanova

**Affiliations:** Kazakhstan Medical University “KSPH”, Almaty, Kazakhstan; Novosibirsk State Medical University, Novosibirsk, Russia; Asfendiyarov Kazakh National Medical University, Almaty, Kazakhstan

**Keywords:** Prediction, Preterm birth, Risk factors of preterm birth

## Abstract

Preterm birth (PTB) remains a significant challenge in modern obstetric practice, posing considerable risks to maternal and neonatal health. Despite advancements in medical technology, the incidence of PTB remains high, and its prediction continues to be complex. Traditional methods for predicting PTB, including medical history evaluation, cervical length measurement, and biochemical markers, have shown limited precision in preventing this serious complication. However, recent technological advancements—such as machine learning algorithms, biomarker profiling, and genetic analyses—offer new possibilities for improving prediction accuracy. This review critically examines current and emerging approaches for PTB prediction, highlighting their potential to transform early risk detection. It also addresses the ethical and societal implications of these technologies. This narrative review aims to comprehensively analyse contemporary methods for predicting preterm birth, evaluating established and emerging approaches. It will assess the efficacy of current predictive tools, examine the limitations they face, and explore the potential for integrating advanced technologies to improve outcomes. By highlighting recent developments in the field and addressing critical knowledge gaps, this review seeks to contribute to the ongoing effort to enhance PTB prediction, aiming to improve maternal and neonatal health outcomes. The study’s novelty lies in its comprehensive analysis of cutting-edge innovations in PTB prediction and its focus on identifying critical gaps in current practices.

## Introduction

Preterm birth (PTB), defined as delivery before 37 completed weeks of gestation, is a critical public health concern globally. According to international medical statistics, PTB occurs in 5% to 18% of all pregnancies, with significant variation across different regions and socioeconomic conditions [1,2,3]. It is a leading cause of neonatal mortality and ranks second in mortality rates among children under five years of age [4]. Each year, over 15 million infants are born preterm, and they face a myriad of short- and long-term health risks. In the immediate neonatal period, preterm infants are particularly vulnerable due to their underdeveloped immune systems, making them susceptible to infectious complications such as pneumonia, meningitis, and sepsis [5]. Furthermore, organ immaturity predisposes these newborns to serious complications, including intraventricular hemorrhages, necrotising enterocolitis, and respiratory distress syndrome, all of which can lead to high neonatal morbidity and mortality. Long-term sequelae, such as cerebral palsy, developmental delays, and sensory disorders (including vision and hearing impairments), are prevalent in preterm survivors [6].

The effects of PTB extend beyond infancy, affecting the individual’s lifelong health. Children born prematurely often experience learning difficulties, cognitive impairments, and behavioral disorders [7]. Moreover, there is growing evidence of a correlation between preterm birth and the development of chronic diseases later in life, particularly cardiovascular conditions and type 2 diabetes mellitus [8]. These findings highlight the multifaceted challenges of PTB, emphasising its immediate clinical implications and its long-term impact on public health.

From a socio-economic perspective, PTB imposes a significant financial burden on healthcare systems globally. The high costs associated with intensive neonatal care, extended hospital stays, and specialised medical interventions, coupled with the need for long-term rehabilitation, educational support, and social services, contribute to this economic strain [9]. To effectively predict preterm birth (PTB), it is crucial first to identify the key risk factors that contribute to its occurrence. Understanding these factors allows for a more accurate assessment of high-risk pregnancies and the development of targeted predictive models (Table 1).

**Table 1.** Major Risk Factors for Preterm Birth

**Category**	**Specific Risk Factors**
Maternal Factors	Advanced maternal age, high/low BMI, smoking, previous PTB, infections (e.g., H. pylori, systemic lupus erythematosus), chronic diseases (hypertension, diabetes).
Fetal Factors	Multiple gestations, fetal growth restriction, and high fetal fibronectin levels.
Cervical/Uterine Factors	Short cervical length, cervical insufficiency, history of cervical surgeries (e.g., cone biopsy), uterine anomalies (fibroids, adenomyosis).
Socioeconomic & Environmental	Low socioeconomic status, poor nutrition, high stress levels, and exposure to pollutants.

Given PTB’s complex and multifactorial nature, various predictive methods have been developed to assess the likelihood of preterm delivery. These methods range from traditional clinical assessments to advanced machine learning algorithms. Each approach has its strengths and limitations, influencing its effectiveness in real-world clinical practice (Table 2).

**Table 2.** Current and Emerging PTB Prediction Methods

**Method Type**	**Examples**	**Limitations**
Clinical History-Based	Prior PTB, maternal age, obstetric history	Limited predictive power, does not account for new risk factors.
Biomarker-Based	Cervical length measurement, fetal fibronectin, and inflammatory markers	Requires standardisation, variability in results.
AI/Machine Learning	Predictive algorithms (QUiPP, CLEOPATRA), deep learning models	Data quality issues, algorithm biases, ethical concerns.

This review aims to comprehensively analyse contemporary methods for predicting preterm birth, evaluating both established and emerging approaches. It will assess the efficacy of current predictive tools, examine their limitations, and explore the potential for integrating advanced technologies to improve outcomes. By highlighting recent developments in the field and addressing critical knowledge gaps, this review seeks to contribute to the ongoing effort to enhance PTB prediction, aiming to improve maternal and neonatal health outcomes.

## Materials and Methods

**Figure 1.**
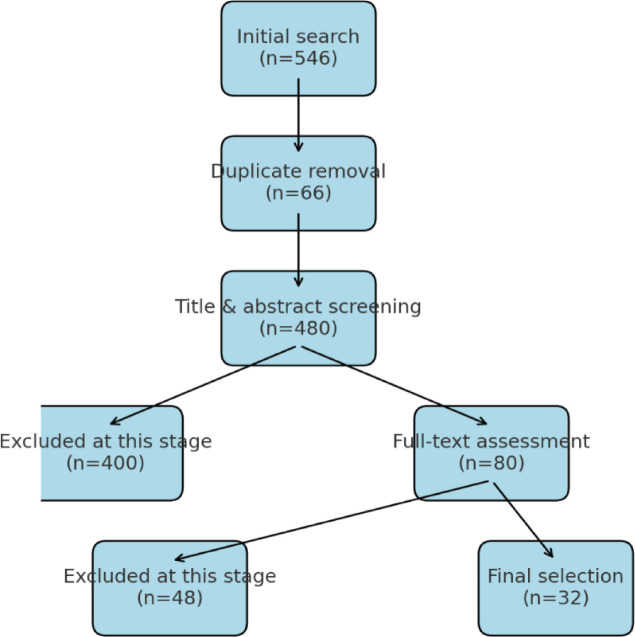
Stages of selection of publications for inclusion in the review.

This review critically analyses recent advances in the field of preterm birth prediction (PTB) based on a comprehensive and methodologically sound literature search. The search was conducted in NCBI (PubMed), Scopus, Medline, and Google Scholar, covering publications from 2019 to 2024. Logical operators and MeSH terms were used to ensure the accuracy and reproducibility of the search. In particular, the search queries were: PubMed: (“premature birth” [Grid] And “forecasting” [All fields]) AND (“machine learning”, OR “biomarker profiling”, OR “genetic analysis”) Scopus: THE NAME IS ABS-KEY (“premature birth” And “forecasting” And (“artificial intelligence” OR “biomarkers”)) Medline: (“premature birth” And “prognosis” And “cervical length measurement”) AND (“risk factors” OR “biomarkers”) Google Scholar: “the prognosis of premature birth” + “artificial intelligence” + “biomarkers” Strict inclusion and exclusion criteria were followed during the research selection process. A careful selection was carried out from the extensive initial list of articles to identify the most relevant and high-quality studies (n=546). The large volume of publications reflects the breadth of research in the field of PTB forecasting. After deleting 66 duplicates, 480 articles were checked by title and annotation. At this stage, 400 articles were excluded due to inconsistent PTB forecast, focus on unrelated results, or lack of methodological detail. The most common reasons for exclusion were articles on therapeutic interventions without predictive models, conference abstracts without a complete methodological description, and research beyond the review period. Subsequently, 80 full-text articles were subjected to detailed verification. Studies were excluded if they contained outdated methodologies, insufficient sample size, or excessive risk of bias. Ultimately, 32 papers were selected for inclusion due to their scientific rigor and direct contributions to developing PTB prediction models, with a particular focus on new technologies such as machine learning and biomarker profiling (Figure 1).

The data was extracted independently by two experts using a standardised approach and clear selection criteria to ensure objectivity and reproducibility. The discrepancies were resolved by consensus. Although this is a descriptive review, a qualitative assessment of the included studies has been conducted, focusing on their methodology, strengths, limitations, and clinical relevance. This approach has provided a scientific synthesis that highlights both progress and challenges in the field of TB prediction research.

## Results

The challenges in accurately predicting preterm birth highlight the need for robust risk stratification systems. A systematic review conducted by Amaro Ferreira from Portugal focused on identifying existing risk scoring systems (RSSs) for PTB, elucidating their constituent variables, and evaluating their performance [10]. Through thorough searches of two comprehensive databases and independent screening and eligibility assessments by two authors, 56 relevant studies were included. The most integrated variables within the examined RSSs included maternal age, weight, history of smoking, previous PTB, and cervical length. However, the performance metrics varied considerably across studies, with sensitivity ranging from 4.2% to 92.0% and the area under the curve (AUC) from 0.59 to 0.95. Despite advancements in technology and scientific understanding, RSSs continue to demonstrate limited predictive capability for PTB, posing challenges for their integration into routine clinical practice.

The study concludes by advocating for the development of new RSSs, the identification of additional variables associated with PTB, and the establishment of a comprehensive reference dataset to address this issue.

In a meta-analysis and systematic review conducted by Romero and colleagues, it was observed that the use of vaginally administered progesterone preparations effectively reduces the incidence of PTB by 41%, while also decreasing neonatal mortality and common neonatal complications such as respiratory distress syndrome, intraventricular hemorrhage, necrotizing enterocolitis, and neonatal sepsis [8]. Other studies, including a meta-analysis by Z. Jin et al., have confirmed the beneficial impact of using an obstetric discharge pessary to prevent PTB. This technique can potentially reduce the incidence of tocolysis and cesarean delivery without increasing the risk of premature rupture of membranes or compromising perinatal outcomes [9]. Findings from a systematic review conducted by R. Brown et al. affirmed the efficacy of cervical cerclage in pregnant women with a history of preterm labor and a cervix shorter than 25 mm [11]. These studies support the use of vaginal progesterone, obstetric discharge pessary, and cervical cerclage as effective interventions for PTB prevention. These measures are recommended for high-risk expectant mothers and help prevent potential PTB occurrences, thereby reducing unnecessary hospitalizations and medical interventions.

Multicenter investigations have delineated primary determinants of PTB, including body mass index (BMI), age, parity, blood pressure, multiple gestations, early onset of sexual activity, and previous PTB history [7] Additionally, factors such as prior wedge-shaped cervical biopsies shortened cervical length [5], cervical biomechanical properties, uterine fibroids and adenomyosis, and elevated fetal fibronectin concentrations [12] are also significant. Other factors that may increase PTB risk include infections, gastroesophageal reflux disease (GERD), Helicobacter pylori infection, systemic lupus erythematosus, and hydroxychloroquine use [13]. Despite efforts to assess PTB risk and develop effective preventive strategies, predicting and preventing PTB remains a formidable challenge. Substantial reductions in PTB frequency have remained elusive over many years. Various tools have been developed to address this problem, including calculators, mobile applications, and assessment scales. For instance, the Fetal Medicine Foundation’s calculator is designed to evaluate risk based on medical history and cervicometry results [14]. The QUiPP mobile app, developed by King’s College London researchers, offers a quantitative assessment of PTB risk, incorporating obstetric history, history of isthmic-cervical insufficiency correction, cervicometry, and fetal fibronectin concentration [15]. Additionally, the CLEOPATRA I and CLEOPATRA II PTB prognosis scales, devised by I. Tekesin et al., are based on factors such as maternal age, obstetric history, cervicometry data, and fetal fibronectin levels [16]. Studies dedicated to PTB prediction are limited by the assumption that all other factors, apart from those under investigation, remain constant. However, in real clinical scenarios, these conditions may vary, reducing forecast accuracy. Improving prediction precision requires incorporating additional features into algorithms and considering their intricate relationships for prognostication. This objective can be achieved through the application of machine learning techniques.

Traditional methods, such as cervical length measurement and fetal fibronectin testing, have been foundational in PTB prediction. However, these methods often exhibit limited sensitivity and specificity, particularly in diverse populations. For instance, cervical length as a predictor may not be universally applicable due to variations in cervical anatomy across different ethnic groups. Moreover, these methods are frequently influenced by external factors such as the timing of testing and patient compliance, further limiting their predictive value.

The convergence of medical science breakthroughs with modern information technologies, such as artificial intelligence, cloud computing, and big data analysis, enhances the accuracy of predicting adverse pregnancy outcomes [17,18,19,20]. Consequently, healthcare practitioners can proactively avert complications and implement timely preventive measures. Intelligent algorithms play a pivotal role in assessing the risk of preterm birth, with research in this domain being conducted in medical centers across numerous countries. For example, I. Vovsha and colleagues from the Center for Computing Systems at Columbia University (USA) conducted a study utilizing artificial intelligence algorithms to estimate the risk of PTB based on data from 2,929 women. Comparing the sensitivity and specificity of logistic regression with the support vector method revealed that the latter exhibited the highest sensitivity, reaching 60%. Furthermore, researchers identified risk factors such as socioeconomic status, race, and maternal age [17].

Similarly, a study by A.-G. Malea et al. at the Polytechnic University of Timisoara (Romania) focused on predicting PTB using machine learning techniques. Leveraging data from 546 medical records from Timisoara Hospital, the researchers conducted several steps, including data preprocessing, clustering, and classification. They identified risk factors such as smoking, maternal age over 35, and low education level, achieving a prediction accuracy of 88% using the naive Bayesian classifier [21]. These investigations underscore the potential of intelligent algorithms in forecasting PTB risk and their significance in advancing medical practice.

A study conducted by A. Koivu and M. Sairanen from the University of Turku in Finland (Faculty of Future Technologies) aimed to evaluate the effectiveness of machine learning (ML) algorithms [14]. The research utilized data from nearly 16 million observations from the US Centers for Disease Control and Prevention (CDC) and the Department of Health and Mental Hygiene (NYC), gathered over three years. Twenty-six predictors of stillbirth and other ailments were identified, and correlation analyses were conducted. Following data cleaning and preparation, including inclusion criteria, markup, and standardization, 70% of the data array was allocated for algorithm training, 10% for model building, and 10% for efficiency assessment. The ML algorithms employed included artificial neural networks (ANN), decision trees with gradient boosting, and algorithm ensembles. Logistic regression served as a comparative method. Among the predictors of preterm birth, diabetes, arterial hypertension, a history of PTB, infertility treatment, assisted reproductive technologies, and marital status demonstrated prognostic significance. The study revealed the high efficacy of ML methods, with an Area Under the Curve (AUC)я of 0.64 for PTB. This metric significantly surpassed results obtained using standard statistical methods, underscoring the superior predictive capability of ML algorithms. The AUC, ranging from 0 to 1, indicates model performance, with 1 representing perfection and 0.5 indicating random guessing. An AUC of 0.64 suggests the model adequately distinguishes between women at risk of PTB and those not at risk, though opportunities for refinement exist [14, 22].

Another study, conducted by H.-Y. Chen and colleagues from the National Taiwan University aimed to assess risk factors for PTB using an ANN and a decision tree [22]. Data from 910 mother-child pairs from the National Register of Taiwan were utilised. The study encompassed the collection and preparation of data, incorporating various parameters such as maternal age, education, occupation, height, weight, lifestyle, and income level. The dataset was partitioned into two segments: 50% for model training and 50% for testing. Through analysis employing an artificial neural network, the study identified the 15 most influential predictors impacting the risk of preterm birth. The ANN exhibited high accuracy, ranging from 80% to 100% across various risk factors. Additionally, a decision tree method was utilised for data categorisation. Results revealed that factors including multiple pregnancies, antepartum bleeding, maternal age, presence of gynecological conditions, and history of preterm birth exerted the most substantial influence on preterm birth development. Furthermore, pre-pregnancy body weight, maternal alcohol consumption and smoking, as well as paternal lifestyle factors such as smoking and alcohol consumption, were identified as significant risk factors for preterm birth (Table 3).

**Table 3.** Overview of Studies on Artificial Intelligence and Machine Learning Methods in Preterm Birth Prediction

**Study**	**Authors**	**Sample Size**	**Methods**	**Key Risk Factors Identified**	**Prediction Accuracy**	**Findings/Results**
Risk of Preterm Birth Prediction	I. Vovsha et al. Columbia University (USA)	2,929 women	AI algorithms (logistic regression vs. SVM)	Socioeconomic status, race, maternal age	SVM: 60% sensitivity	SVM outperformed logistic regression in sensitivity for PTB prediction.
Predicting PTB with Machine Learning	Polytechnic University of Timisoara (Romania)	546 records	Machine learning (naive Bayesian classifier)	Smoking, maternal age >35, low education level	88% accuracy	Achieved high prediction accuracy using a naive Bayesian classifier.
Evaluating Machine Learning Algorithms for PTB	A. Koivu & M. Sairanen University of Turku (Finland)	16 million data points	ML algorithms (ANN, decision trees, ensembles)	Diabetes, hypertension, PTB history, infertility treatment, and marital status	AUC 0.64	ML methods showed superior predictive ability, significantly outpacing traditional statistical methods.
Assessing PTB Risk Factors with ANN	H.-Y. Chen et al. National Taiwan University	910 mother-child pairs	ANN, decision tree	Multiple pregnancies, antepartum bleeding, maternal age, and gynecological conditions	80–100% accuracy (ANN)	Identified 15 influential risk factors for PTB, with ANN providing high accuracy in prediction across various risk factors.

While machine learning methods in medicine hold considerable potential, they encounter limitations [23, 24]. Ethical considerations, particularly regarding patient data confidentiality, necessitatethe development of depersonalized data versions [25]. Additionally, data quality from medical records may vary, posing challenges to processing; however, natural language text processing by computers can help mitigate this issue [26]. Unreliable data sources and improper model selection for training may lead to algorithm overfitting or underfitting. Moreover, the “AI chasm” phenomenon suggests that even high-quality algorithms can be ineffective when applied to low-quality data in real clinical settings. Addressing these limitations requires a cautious approach to standards and technology development to maximise the potential of machine learning in medicine [27,28,29,30,31,32].

Among the various AI techniques, support vector machines (SVM), random forests, and neural networks have shown promise in PTB prediction. SVMs are valued for their ability to classify complex datasets with high-dimensional spaces, while random forests effectively handle large, heterogeneous data by aggregating the results of multiple decision trees [33,34,35]. Neural networks, particularly deep learning models, can learn intricate patterns within vast datasets, making them highly suitable for predicting PTB based on a combination of clinical, genetic, and imaging data. A critical concern in applying AI to PTB prediction is the potential for algorithmic bias, particularly regarding racial and socioeconomic disparities [36,37,38,39,40]. AI models trained on datasets that predominantly represent specific populations may not generalise well to other groups, leading to inaccurate predictions for underrepresented demographics. Addressing these biases requires careful consideration of training data diversity and ongoing validation across diverse population subsets.

This section concludes with a comparative table that provides a concise overview of the results obtained from some traditional and cutting-edge technologies used in predicting preterm birth (PTB). Building upon the discussion on potential biases and the significance of diverse data, this table clearly synthesises the predictive methods employed, their key indicators, and their performance outcomes (Table 4).

**Table 4.** Traditional and cutting-edge technologies used in predicting preterm birth

**Technology**	**Study (Year)**	**Key Predictive Factors**	**Sample Size**	**Sensitivity (%)**	**Specificity (%)**	**AUC**
Risk Scoring Systems (RSS)	Ferreira et al. (2023)	Maternal age, smoking, cervical length	56 studies (Meta-analysis)	4.2–92.0	Varies	0.59–0.95
Vaginal Progesterone	Romero et al. (2018)	Cervical length, prior PTB	Meta-analysis	41% reduction in PTB	—	—
Obstetric Pessary	Jin et al. (2019)	Cervical insufficiency	Meta-analysis	Reduced cesarean rates	—	—
Cervical Cerclage	Brown et al. (2019)	Short cervix (<25 mm)	Systematic review	Effective in high-risk women	—	—
QUiPP Mobile App	Lee et al. (2020)	Cervicometry, fibronectin	Application-based cohort	High	High	—
Machine Learning (ANN)	Chen et al. (2011)	Age, BMI, smoking, bleeding	910 (Taiwan cohort)	80–100	—	—
SVM (AI Model)	Vovsha et al. (2014)	Socioeconomic status, maternal age	2,929 (USA)	60	75	0, 70
Naive Bayesian Classifier	Malea et al. (2010)	Smoking, age >35, low education	546 (Romania)	88	82	0, 85
Ensemble ML Models	Koivu & Sairanen (2020)	Diabetes, hypertension, IVF history	16 million (CDC)	—	—	0, 64

## Discussion

The analysis of traditional and advanced methodologies for predicting preterm birth (PTB) underscores notable disparities in diagnostic performance and predictive reliability. Classical approaches, exemplified by cervical length measurement and fetal fibronectin assessment, while foundational in obstetric practice, demonstrate pronounced variability in sensitivity and specificity. Ferreira et al. [10], through an extensive meta-analysis encompassing 56 studies, delineate substantial fluctuations in sensitivity from 4.2% to 92.0%, thereby highlighting the limited universal applicability of traditional risk scoring systems within diverse patient cohorts. This inconsistency underscores the necessity for novel, integrative methodologies to deliver more precise, individualised risk stratifications.

Artificial intelligence (AI) and machine learning (ML) advancements have yielded transformative opportunities in PTB prediction. Investigations conducted by Vovsha et al. [16] and Malea et al. [20] underscore the superiority of AI algorithms over conventional logistic regression models, particularly when analysing multidimensional socioeconomic and behavioral determinants. Malea et al. illustrate the efficacy of ML techniques in identifying latent predictors, such as tobacco use and educational attainment, which frequently elude traditional analytical frameworks. Despite the notable predictive gains, Vovsha et al. caution against the overreliance on training datasets derived from resource-abundant environments, thereby exposing the risk of diminished model validity across heterogeneous population segments.

Nonetheless, significant challenges persist in the translational application of AI technologies. Koivu and Sairanen [14] accentuate the limited generalizability of ML-driven models, attributing this deficiency to the narrow demographic scope of training datasets, which often fail to encapsulate the complexities of underrepresented ethnic and socioeconomic groups. Additionally, Bhunia et al. [26] articulate the concept of the ‘AI chasm,’ wherein models exhibiting robust performance under experimental conditions falter when confronted with the multifactorial variability of real-world clinical environments.

Equally critical are algorithmic deployment’s ethical and methodological implications in clinical practice. Chen et al. [22] emphasise the perils of model overfitting, a phenomenon exacerbated by imbalanced datasets that skew predictive outputs and compromise clinical decision-making integrity. Furthermore, the opaque nature of algorithmic operations exacerbates concerns regarding patient data confidentiality and the propagation of algorithmic bias, particularly in vulnerable populations.

A comprehensive synthesis of investigations reveals the duality inherent within emerging PTB prediction technologies. Traditional paradigms demonstrate limitations, while advancements in ML integration highlight diagnostic progress and potential. The complexities of algorithmic deployment underscore the importance of rigorous data governance. Advancing the field necessitates a paradigmatic shift integrating obstetric insights with AI methodologies, ensuring predictive accuracy, methodological transparency, and ethical accountability.

We believe ML-based technologies significantly enhance predictive capabilities but cannot fully replace clinical judgment. The most promising approach is integrating traditional clinical indicators with advanced AI techniques to create comprehensive, individualised risk profiles. Additionally, future research should prioritise the development of diverse and representative training datasets to improve model generalizability. Ethical challenges, including algorithmic bias and patient data privacy, must be addressed through rigorous validation and transparent model development practices.

## Conclusions

The results of this review demonstrate that existing methods for predicting preterm birth offer some clinical utility but are limited in their accuracy. Traditional tools such as cervical length measurement and fetal fibronectin testing show low predictive precision across diverse populations, complicating their broader application in routine clinical practice. Innovative technologies, including artificial intelligence and machine learning, exhibit greater efficacy in predicting PTB by processing large datasets and identifying complex interrelations among risk factors. These approaches present promising opportunities for improving predictive outcomes, though their practical implementation necessitates further research to address challenges related to data quality and algorithmic biases. The practical significance of this study lies in the rationale for integrating AI methods to enhance PTB prediction accuracy and develop more reliable screening programs. Future research should focus on adapting existing AI models to real-world clinical settings, expanding the range of predictive variables, and addressing ethical concerns associated with AI applications in healthcare.
